# Exploring Web-Based Information and Resources That Support Adolescents and Young Adults With Cancer to Resume Study and Work: Environmental Scan Study

**DOI:** 10.2196/47944

**Published:** 2024-03-25

**Authors:** Clarissa E Schilstra, Sarah J Ellis, Jennifer Cohen, Alana Gall, Abbey Diaz, Kristina Clarke, Gadiel Dumlao, Jennifer Chard, Therese M Cumming, Esther Davis, Haryana Dhillon, Mary Anne Burns, Kimberley Docking, Eng-Siew Koh, Josephine O'Reilly, Ursula M Sansom-Daly, Joanne Shaw, Nicole Speers, Natalie Taylor, Anthea Warne, Joanna E Fardell

**Affiliations:** 1 Behavioural Sciences Unit, Kids Cancer Centre, Sydney Children's Hospital Faculty of Medicine and Health, Randwick Clinical Campus University of New South Wales Sydney Randwick Australia; 2 Faculty of Medicine and Health Randwick Clinical Campus University of New South Wales Sydney Kensington Australia; 3 Canteen Australia Newtown Australia; 4 National Centre for Naturopathic Medicine Faculty of Health Southern Cross University Lismore Australia; 5 School of Public Health Faculty of Medicine University of Queensland Brisbane Australia; 6 Western Sydney Youth Cancer Service Crown Princess Mary Cancer Centre Westmead Hospital Westmead Australia; 7 Faculty of Arts, Design and Architecture University of New South Wales Sydney Kensington Australia; 8 Disability Innovation Institute University of New South Wales Sydney Kensington Australia; 9 School of Psychology Faculty of Science University of Sydney Camperdown Australia; 10 Faculty of Medicine and Health University of Sydney Camperdown Australia; 11 South West Sydney Clinical School Faculty of Medicine and Health University of New South Wales Sydney Liverpool Australia; 12 Liverpool and Macarthur Cancer Therapy Centres Liverpool Australia; 13 Ingham Institute for Applied Medical Research Liverpool Australia; 14 Cancer survivor representative New South Wales Australia; 15 Sydney Youth Cancer Service Nelune Comprehensive Cancer Centre Prince of Wales Hospital Randwick Australia

**Keywords:** adolescent, cancer, education, employment, information needs, oncology, online information, quality of life, resource, return to work, school, study, supportive resources, treatment, young adult

## Abstract

**Background:**

Adolescents and young adults (AYAs) diagnosed with cancer experience physical, cognitive, and psychosocial effects from cancer treatment that can negatively affect their ability to remain engaged in education or work through cancer treatment and in the long term. Disengagement from education or work can have lasting implications for AYAs’ financial independence, psychosocial well-being, and quality of life. Australian AYAs with cancer lack access to adequate specialist support for their education and work needs and report a preference for web-based support that they can access from anywhere, in their own time. However, it remains unclear what web-based resources exist that are tailored to support AYAs with cancer in reaching their educational or work goals.

**Objective:**

This study aimed to determine what web-based resources exist for Australian AYAs with cancer to (1) support return to education or work and (2) identify the degree to which existing resources are age-specific, cancer-specific, culturally inclusive, and evidence-based; are co-designed with AYAs; use age-appropriate language; and are easy to find.

**Methods:**

We conducted an environmental scan by searching Google with English search terms in August 2022 to identify information resources about employment and education for AYAs ever diagnosed with cancer. Data extraction was conducted in Microsoft Excel, and the following were assessed: understandability and actionability (using the Patient Education and Materials Tool), readability (using the Sydney Health Literacy Laboratory Health Literacy Editor), and whether the resource was easy to locate, evidence-based, co-designed with AYAs, and culturally inclusive of Aboriginal and Torres Strait Islander peoples. The latter was assessed using 7 criteria previously developed by members of the research team.

**Results:**

We identified 24 web-based resources, comprising 22 written text resources and 12 video resources. Most resources (21/24, 88%) were published by nongovernmental organizations in Australia, Canada, the United States, and the United Kingdom. A total of 7 resources focused on education, 8 focused on work, and 9 focused on both education and work. The evaluation of resources demonstrated poor understandability and actionability. Resources were rarely evidence-based or co-designed by AYAs, difficult to locate on the internet, and largely not inclusive of Aboriginal and Torres Strait Islander populations.

**Conclusions:**

Although web-based resources for AYAs with cancer are often available through the websites of hospitals or nongovernmental organizations, this environmental scan suggests they would benefit from more evidence-based and actionable resources that are available in multiple formats (eg, text and audio-visual) and tailored to be age-appropriate and culturally inclusive.

## Introduction

A diagnosis of cancer in adolescence and the young adult years can lead to significant and long-lasting disruptions to key developmental milestones [1-3]. Adolescents and young adults (AYAs) with cancer are at risk of poor long-term medical and psychosocial outcomes due to delays in diagnosis and lagging improvements in survival rates compared with those diagnosed as children or adults [1]. Symptoms and late effects from cancer treatment can negatively impact AYAs’ education and work engagement [4-7]. A weakened immune system, nausea, fatigue, neuropathy, poor cognitive functioning, poor social well-being, and mental health challenges such as depression and social anxiety are just some of the physical symptoms and late effects AYAs must contend with [3-9]. Challenges with education and work engagement may also drive distressing symptoms such as poor social well-being and depression [3,9,10].

Poorer outcomes are exacerbated by the limited age-appropriate services targeting their unique needs [11]. During adolescence and the young adult years, broadly defined as the ages of 15-39 years, young people are expected to participate in and complete education and training, obtain employment, and achieve financial independence, all while navigating social and intimate relationships to develop and evolve their identity [12]. A cancer diagnosis and treatment during adolescence and the young adult years can interrupt or delay these developmental tasks [12].

Extended absences and difficulties engaging with education or work are common for survivors of AYA cancer and can have a lifelong negative impact on AYAs’ educational and work goals, quality of life, and psychosocial and financial well-being [2]. survivors of AYA cancer miss significantly more days of school than their peers [11]. AYAs may miss 40-60 days of school within the first year following their diagnosis [13], and Australian data suggest nearly 50% of survivors of AYA cancer have not fully returned to education or work up to 24 months post diagnosis [14]. Nearly 40% of AYAs report their employment goals were negatively affected by cancer [15], and survivors of AYA cancer are more likely than peers without a history of cancer to report an increased number of missed workdays as a result of illness or disability (11.9% of survivors vs 6.7% of controls) [16]. survivors of AYA cancer are also more likely than peers without a history of cancer to report employment disability (being unable to carry out employment or work requirements at all or needing to do so with disability provisions; 34.1% of survivors vs 23.9% of controls) [16]. However, qualitative studies suggest that survivors of AYA cancer experience trouble navigating public support, education, and employment systems, which puts them at a disadvantage by contributing to increased financial hardship, fear, and uncertainty around their education and employment situations. Financial hardship in itself serves as a barrier to AYAs achieving their education goals by making it difficult for AYAs to afford education, particularly at a university level, or to keep up with repayment of education-related debts [17].

In contrast, AYAs who are able to remain more engaged with their education or work report decreased psychological distress [18] and improved social well-being [1,19]. Yet, few interventions supporting AYAs to remain engaged with education or work have been systematically evaluated [2]. The only such service evaluated in Australia is an educational and vocational counseling service based in a major cancer center in Victoria, Australia, which provides in-depth, tailored support through trained advisors to AYAs diagnosed with cancer between the ages of 15 and 25 years [20]. To date, 209 AYAs have received support through this program. By completion, 73% of AYAs were able to engage in education or vocation or were receiving support through an external source [20]. Access to such personalized programs may be limited due to cancer centers’ resources and AYAs’ distance to their treatment centers [21,22]. Individualized consultation requires synchronous engagement, usually during school or work hours, which may further prevent uptake for AYAs who have some level of participation in education or work [23].

The average Australian AYA spends approximately 14 hours per week on the internet, and survivors of AYA cancer are highly engaged with social media as well as web-based cancer resources [24,25]. Many Australians with cancer living in rural or remote locations rely on web-based resources to navigate the impacts of a cancer diagnosis and its treatment on their education or career [26,27]. Given this reliance on web-based information, it is critical to consider the extent to which web-based information resources are equitably accessible by Australian AYAs. Previous studies and reviews have defined equity of access to web-based information as the provision of web-based information that is easy to find, provided in a range of formats (eg, text, video, and audio), understandable or readable for individuals with varying abilities and health literacy levels, and culturally and linguistically inclusive [28-30]. The importance of equitable access to health information for Australians is paramount, considering how many people live in rural locations and the cultural and linguistic diversity of the country. Approximately 3.2% of the population identify as Aboriginal and Torres Strait Islander peoples, 30% were born overseas, and 21% of families speak a language other than English at home [31].

Ensuring equitable access to web-based information resources requires consultation with target populations (ie, co-design of information resources with a culturally and linguistically diverse group of AYAs diagnosed with cancer), as well as a focus on providing information that is evidence-based. However, no previous research has assessed what web-based information resources exist to support Australian survivors of AYA cancer in their engagement with education or work, and to what extent resources are equitably accessible. Therefore, this study aimed to determine the following: (1) What web-based resources exist for engaging with education or work after a cancer diagnosis that AYAs with cancer are likely to encounter when conducting a Google search? and (2) Of the identified resources, to what degree are they understandable, actionable, readable, easy to locate, evidence-based, co-designed with survivors of AYA cancer, and culturally inclusive?

## Methods

### Overview

Web-based resources for AYAs with cancer are typically provided through hospitals and nongovernmental organizations (NGOs) rather than through academic journals or research databases. Therefore, we chose to conduct an environmental scan rather than a systematic review, using a standard search engine rather than academic databases. Environmental scans have demonstrated usability in identifying health information resources across a range of health disciplines [32,33]. While there is no consensus regarding optimal methods for conducting an environmental scan of health information resources, environmental scans take a higher-level approach than systematic reviews or qualitative evaluation studies to identify available resources, tabulate yes or no responses to whether web-based resources possess certain qualities, and determine the basic usability of resource content [33,34]. We opted to follow similar methods used by Ruble et al [32] in their 2019 publication assessing web-based resources to support children returning to school during or after cancer treatment and methods used by Schiffman et al [35] in their 2006 study on internet use among survivors of AYA cancer. In keeping with these previous studies, 2 researchers led the search and data extraction, and we used validated measures of understandability, actionability, and readability to conduct a basic assessment of available resources. We also tabulated whether resources were easy to locate, evidence-based, developed through co-design with AYAs, and culturally inclusive of Indigenous populations. We conducted structured searches through Google and extracted data in Microsoft Excel (Microsoft Corporation).

### Consumer Involvement

Consumer involvement in the design of this study was central to our methods. Chief investigators included the researcher and clinician chief investigators in addition to 2 survivors of AYA cancer (authors CES and NS) and 1 parent of a survivor (author JO). Together, the chief investigator team met in November 2021, March 2022, and May 2022, to develop the environmental scan protocol, including search terms and methods.

### Searches

We searched Google Australia with English search terms between August 8 and 19, 2022 (Textbox 1). No limits were applied to the country, as we wanted to replicate the way survivors of AYA cancer currently access information to support their return to education or work.

**Environmental scan search terms as per both themes.**

**Adolescents and young adults with or surviving cancer**

- AYA cancer
- Teen cancer
- Adolescent cancer
- Young adult cancer

**Returning to study or work**

- Study
- School
- Education
- University
- College
- Work
- Employment
- Career

Search terms were created by combining search words from 2 themes. The first theme designated the target population of AYAs with or surviving cancer, and the second designated information and resource content related to “returning to study or work.” Using the words listed in Textbox 1, CES and GD independently conducted 24 unique searches combining the 2 groups of search terms with “AND” (eg, “adolescent cancer AND school”). Before and between each new search, the browser cache was reset. All searches were conducted from Sydney, New South Wales, Australia. However, to identify whether there may be any difference in search results based on location in Australia, GD also conducted 6 of the 24 searches using a virtual private network and changed the search location to Perth, Western Australia, which is located on the opposite side of Australia from Sydney.

### Resource Selection

Typically, a Google search will present 10 results per page, meaning 50 results would be presented across 5 pages. Although the average internet user will only click on results appearing in the first 10 Google search results [36], we opted to maximize, the identification of relevant results by reviewing the first 50 results for eligibility (CES and GD) [37]. Eligible websites, documents, videos, and audio-visual resources were those that provided text-based information, video, or audio-visual information in English and were directed primarily toward AYAs returning to study (any level) or work after a cancer diagnosis. Websites, documents, and videos or audio-visual resources targeting parents or family members of AYAs were excluded. Academic papers, media stories, and blogs were also excluded.

### Data Extraction

An Excel spreadsheet was developed to include drop-down menus to record key data (Table 1). Data extraction fields were partially based on a previous review of web-based resources conducted by Ruble et al [32] in 2020.

CES and GD independently conducted data extraction and recorded the addresses of websites meeting eligibility criteria in separate Excel spreadsheets, reconciled their searches, and removed duplicates.

**Table 1:** Data extraction fields and response options.

Field	Response option
Target audience	AYA^a^-specific or other
Setting	Education (inclusive of secondary or tertiary) or employment
Source creator	Nongovernmental organization Cooperative group or professional organization Health care institution State or federal government organizations Media publications
Country of origin	Country name
Access to website	Publicly available, subscription, or user profile
Purpose of website	Information, advertising, support, or intervention
Date of last review of information	Date
Cancer type	Diagnosis name, resource designed for AYAs with chronic illness more broadly but includes cancer, or no cancer type specified
Media used to convey information	Written descriptive text, video or YouTube, images, stories or vignettes, quotes from consumers, or other
Support, tools, or information provided by the resource	Checklists, letter templates, strategies, access to career or education counseling, support group or network, or other
Evidence-based	Yes or no
Co-designed	Yes, no, or unclear: describes consultation with survivors but not methods for this consultation

^a^AYA: adolescent and young adult.

### Assessment of Resources and Data Synthesis

#### Was the Resource Understandable and Actionable?

For both text and audio-visual resources, we used the Patient Education Materials Assessment Tool (PEMAT) [38] to assess the understandability and actionability of the resources. Understandability refers to whether the meaning is comprehensible, taking multiple elements into account, such as word complexity and the layout or structure of the information [38]. Actionability refers to whether or not a resource provides content in a way that consumers can easily determine what they need to act on or do based on the content presented [38]. PEMAT for written materials consists of 17 items assessing understandability and 17 items assessing actionability, all of which are scored as agree, disagree, or unsure [38]. The PEMAT for audio-visual materials includes 13 items assessing understandability and 4 assessing actionability. The PEMAT generates percentage scores (0%-100%) which is the proportion of the responses assessed as having been met (agree). Scores of 100% indicate optimal understandability or actionability; scores of 70% indicate adequate understandability or actionability [38,39]. The PEMAT has been used previously in a review of information resources for students with cancer [32] and an evaluation of other web-based information for many illnesses, including cancer [37]. The PEMAT demonstrates good reliability and ease of use, with interrater reliability scores of 0.92 for understandability and 0.93 for actionability, and 92% of raters agreeing on its ease of use [40].

#### Was the Resource Readable?

We also assessed the readability (reading level) of text resources using the Sydney Health Literacy Laboratory (SHeLL) Health Literacy Editor. Optimal readability on the SHeLL Editor is indicated by a score of 8 or below, equating to a grade 8 reading level [41,42]. Generally, health information designed for the general population or patients is recommended to be readable at a grade 8 level or lower [43]. The SHeLL Editor enables the pasting of exact text from a resource into its reading level calculator to provide a specific reading level for text.

#### How Easy Was It to Locate the Resource?

The ease of locating the resource was assessed by determining whether a resource appeared within the first 10 search results on the first page of results on Google. This is based on evidence suggesting the average internet user will only click on results appearing in the first 10 Google search results [36].

#### Was the Resource Evidence-Based?

Resources were evaluated as being evidence-based according to whether or not supporting evidence was cited and accurately represented to support the information they provided, or if they indicated in any background content whether research was involved in the development of the resource content.

#### Was the Resource Developed Through Co-Design With Survivors of AYA Cancer?

Co-design refers to methods used to engage, consult, and work in collaboration with young people to develop research questions, resource content, or interventions [44]. We assessed whether resources were co-designed with survivors of AYA cancer based on whether or not they described using a co-design method to develop the information they provide. We note as “unclear” any resources indicating content was developed in consultation with AYAs with cancer, but the exact co-design methods used or extent of engagement with AYAs is not clearly described.

#### Was the Resource Culturally Inclusive?

To our knowledge, no tool exists to assess the cultural inclusivity of international web-based health resources. Therefore, to measure the cultural inclusivity of the resources we identified, we used 7 criteria that were codeveloped by 3 Aboriginal and Torres Strait Islander researchers (including AG) and 2 non-Aboriginal and Torres Strait Islander researchers (including AD) [45]. All researchers involved in the development of these criteria have a strong track record in Indigenous health research [45-51]. Due to the criteria being designed solely for the evaluation of the cultural inclusivity of resources for Aboriginal and Torres Strait Islander peoples in Australia, we only evaluate the cultural inclusivity of resources created by and for Australians [45].

The criteria for cultural inclusivity were as follows [45]: (1) Does the resource include any visual aids (photos, animations, infographics, or charts) that depict or contain information about Aboriginal and Torres Strait Islander peoples? (2) Does the resource include any information or data about Aboriginal and Torres Strait Islander peoples? (3) Does the resource include any Aboriginal and Torres Strait Islander design or artwork? (4) Does the resource provide any evidence of leadership, involvement, or governance by peoples, communities, or organizations that identify as or represent populations that are Aboriginal and Torres Strait Islander? (5) Is the resource available in any Aboriginal and Torres Strait Islander languages? (6) Is any of the language used strengths-based and respectful to Aboriginal and Torres Strait Islander peoples? and (7) Does the resource include a contact (phone number, email, or website) for any culturally relevant or personalized support and information for Aboriginal and Torres Strait Islander peoples?

AG, a Pakana woman from Lutruwita (Tasmania), reviewed all resources to determine their relevancy to Aboriginal and Torres Strait Islander peoples. As this tool is not validated, there is no clear minimum number of criteria that should be achieved to determine the cultural competency of a resource. As such, we report the number of criteria that were met descriptively and describe the strengths and shortfalls of the resources.

## Results

### Research Question 1: What Web-Based Resources Exist for Engaging With Education or Work After a Cancer Diagnosis, That AYAs With Cancer Are Likely to Encounter When Conducting a Google Search?

A total of 24 AYA-specific resources met eligibility criteria and were included (Table 2). Most were published by NGOs (n=19, 79%). All resources focused on information provision rather than advertising, support, or intervention, with content shared through text information, text and video stories from other AYAs with cancer, contact details for support organizations, or lists of strategies to navigate education and work challenges. A total of 8 resources were from the United States [52-59], 8 from Australia [60-67], 6 from the United Kingdom [68-73], and 2 from Canada [74,75]. A total of 7 resources focused on education, 8 on work, and 9 on both. There was no difference in search results between searches conducted in Sydney and Perth.

Most resources did not target specific cancer types or stages of the cancer trajectory, although 4 were developed for people diagnosed with blood cancer [59,66,69,74]. There was little consistency in the topics covered across resources, with only a few common topics covered (Table 3).

**Table 2:** Cultural inclusivity of resources identified through the environmental scan [45].

Criteria	Resources meeting each criterion, n
Does the resource include any visual aids (photos, animations, infographics, or charts) that depict or contain information about Aboriginal or Torres Strait Islander peoples?	0
Does the resource include any information or data about Aboriginal or Torres Strait Islander peoples?	0
Does the resource include any Aboriginal or Torres Strait Islander designs or artwork?	4
Does the resource provide any evidence of leadership, involvement, or governance by people, communities, or organizations that identify as or represent populations that are Aboriginal or Torres Strait Islander?	0
Is the resource available in Aboriginal or Torres Strait Islander languages?	0
Is any of the language used strengths-based and respectful to Aboriginal and Torres Strait Islander peoples?	6
Does the resource include a contact (phone number, email, or website) for any culturally relevant or personalized support and information for Aboriginal and Torres Strait Islander peoples?	2

**Table 3:** Common information and strategies covered in resources.

Topic covered	Resource explored topic in relation to education, work, or both
Disability rights and accessing accommodations or provisions (n=16) [52-55,58,61-63,65-67,71,72,74]	Both
Telling people or talking about your cancer (n*=*13) [52-55,60-63,65-67,70-72]	Both
Applying for jobs or changing careers (n=11) [52,61,64,66,67,69,71-75]	Both
Managing physical or cognitive impacts of cancer treatment (n=7) [54,57,65-67,71,75]	Both

### Research Question 2a: Were Resources Understandable?

Understandability of all text and resources was very good, with all but 1 resource [68] scoring 80% or more on the PEMAT (Table S1 in Multimedia Appendix 1 [52-75]).

### Research Question 2b: Were Resources Actionable?

Actionability varied greatly across resources, ranging from 40%-100% on the PEMAT (Table S1 in Multimedia Appendix 1 [52-75]). Less actionable text resources tended to be those focused on broad information and strategies, such as tips on how to tell your employer about your diagnosis and the suggestion to seek counseling support through a university campus student services center, rather than advice on when and how AYAs can take specific steps to address their concerns. In general, video resources were the least actionable in that most involved AYA survivors telling their own personal stories related to education and work challenges after a cancer diagnosis, rather than providing advice or strategies to other AYAs.

### Research Question 2c: Were Text Resources Also Readable?

Readability for all 23 text resources was very poor, with reading levels ranging between grades 8.5 and 16.0 (mean 12, SD 1.97), indicating that, on average, the included resources require completion of a high school degree to comprehend. No resources were assessed to be the optimal reading level of grade 8 or lower (Table S1 in Multimedia Appendix 1 [52-75]).

### Research Question 2d: Were Resources Easy to Locate?

Resources were difficult to locate, with 22 out of 24 (90%) relevant resources appearing on the second or third page of the Google search results (Table S1 in Multimedia Appendix 1 [52-75]). Results of the Google search prioritized resources related to younger children diagnosed with cancer and their engagement with school, as well as older adults returning to work. AYA-specific resources were scattered in between these less relevant results, as well as other, less relevant results, such as academic journal publications, links to hospital-based cancer services, and information about specific types of cancer.

### Research Question 2e: Were Resources Evidence-Based?

No resources cited any research-based evidence to support the information provided (Table S1 in Multimedia Appendix 1 [52-75]). Most resources did not describe how the content was developed. Where any description was provided, resources tended to be developed through consultation with expert informants, such as career counselors, oncologists, hematologists, and social workers.

### Research Question 2f: Were Resources Co-Designed?

No resources specifically discussed co-design methods used to develop resource content in collaboration with AYAs.

### Research Question 2g: Were Resources Culturally Inclusive?

The number of cultural inclusivity criteria each resource addressed is summarized in Table S1 in Multimedia Appendix 1 [52-75]. The number of resources meeting specific criteria in the cultural inclusivity checklist is summarized in Table 2. Generally, cultural inclusivity of Aboriginal and Torres Strait Islander peoples was very poor, with only 2 out of 7 inclusivity criteria met by 1 or more resources: inclusion of Aboriginal or Torres Strait Islander cultural design or artwork and an acknowledgment on the web page recognizing Aboriginal and Torres Strait Islander peoples (minimally reflecting the use of strengths-based language that is respectful of Aboriginal and Torres Strait Islander peoples).

In reviewing all resources, both Australian and internationally-designed, we noticed several further equity, access, diversity, and representative issues with resources that were not initially part of our aims, that are important to highlight. Pictures and videos presented in resources almost exclusively portrayed heterosexual relationships, women were more commonly represented in pictures than men, and women were more commonly shown to be young, White, and of thinner build. Settings also showed middle-class, suburban, or urban areas rather than lower-socioeconomic, rural, or remote settings. Most resources also primarily assumed internet access and support from family or friends were available to AYAs. Lastly, language was not gender neutral and tended to assume heterosexual, 2-parent families.

## Discussion

### Overview

This environmental scan aimed to (1) determine what web-based resources exist to support survivors of AYA cancer in their engagement with education or work and (2) assess the understandability, readability, actionability, and cultural inclusivity of resources, as well as how easy resources were to locate, whether they provide evidence-based information, and whether they were co-designed with survivors of AYA cancer. We found few high-quality resources on the topic of returning to education or working for AYAs with cancer in Australia.

Although the understandability of most resources was high, the readability of text-based resources was poor, with most text resources requiring reading levels at the university education level or higher. This discrepancy may be due to the understandability criteria being quite broad (eg, text “material uses common, everyday language” or “material ‘chunks’ information into short sections”) and not directly providing criteria against which age-appropriate understandability could be assessed. For example, a resource might include everyday language for a young adult in small sections, but sentence structures or legal terminology related to education or work rights may be more complicated, thus affecting readability. Our findings on poor readability of resources are consistent with literature indicating most AYAs with cancer who access web-based resources report the resources require high health literacy and present information that is difficult to understand, critically evaluate, and act on [76,77].

We also found most web-based resources limited in their modes of information provision, primarily using text to provide lists of information and strategies. Few resources involved audio-visual content that may be preferable for the AYA population [35]. Where audio-visual content was provided, it was often focused on individual stories and experiences rather than the provision of guidance to AYAs and actionable strategies to navigate the return to education or work after cancer.

It is therefore unsurprising that the actionability of resources was moderate, with resources scoring 60%-100% on actionability. The focus on broad information and strategies in most of the resources reviewed may feel overwhelming to AYAs, given that it can be difficult for a young person to review a long list of suggestions and determine what they should act on given their individual health status, needs, and education or work goals [25]. Previous studies have provided evidence that Australian survivors of AYA cancer report low confidence in their ability to assess the reliability and validity of health-related information [77]. From a developmental standpoint, adolescents may not have fully developed their critical thinking skills yet [77]. This underscores the importance of providing information to AYAs that is both understandable and actionable.

It is also important to note that no resources cited an evidence base (ie, peer-reviewed scientific literature) for their information or recommendations, nor did any resource specify co-design of any content with survivors of AYA cancer. Instead, most resources assumed a certain level of understanding and self-motivation to act on the provided information or strategies. Most resources were also difficult to locate, appearing on multiple pages in a Google search. However, reliance on evidence-based information and co-design of resources with AYA survivors are widely acknowledged as critical to ensuring that information content and delivery methods are optimized for the specific needs of this age group [78-81].

Lastly, few resources met more than 1 criterion for cultural inclusivity. The lack of culturally inclusive resources for Aboriginal and Torres Strait Islander peoples may exacerbate existing health inequalities in people with cancer [82]. While there is no research, to our knowledge, describing specific concerns related to return to work or education for Aboriginal and Torres Strait Islander AYAs with cancer, financial distress is a common area of unmet need for Aboriginal and Torres Strait Islander adults with cancer in Queensland [83]. Considering the employment and educational disparities that are known within this population broadly [84,85], it stands to reason that Aboriginal and Torres Strait Islander AYAs with cancer may be in particular need of information to support their educational and employment endeavors. However, resources were generally not inclusive of Aboriginal and Torres Strait Islander peoples. In turn, the literature suggests that such limited inclusivity in health information can lead to feelings of isolation, feeling misunderstood by health services, and reduced self-efficacy in patients to follow medical advice [86]. There is an urgent need to address this gap in resources available to Australian AYAs through co-designing and testing the impact of resources with Aboriginal and Torres Strait Islander survivors of AYA cancer.

### Comparison to Previous Work

While some NGOs and health care institutions provide lists of web-based resources for AYAs with cancer on their website, no previous research has specifically collated and evaluated web-based resources to support AYAs’ return to education or work after cancer. This environmental scan is the first to evaluate the age appropriateness, accessibility, understandability, and cultural inclusivity of web-based resources specifically targeting the education and work needs of AYAs with cancer in Australia.

### Limitations

There were some limitations worth noting. The search strategies used were constructed to mirror typical searches AYAs might conduct with a select set of keywords. However, these strategies may not capture all modes of searches AYAs might conduct, such as asking questions in the Google search or using other terms not featured, and we did not include results such as blogs or social media posts from which AYAs may also seek information. We only conducted searches in English and did not find or include resources published in other languages that may be relevant. Furthermore, there are some limitations associated with assessing the cultural inclusivity of international resources using a tool designed specifically for Australia. However, it is important to note that, to our knowledge, no international tools exist to assess the cultural inclusivity of web-based health information resources, and we, therefore, opted to use the Australian tool and focus specifically on assessing the cultural inclusivity of resources for Aboriginal and Torres Strait Islander Australians. We also aimed to optimize the reach of our searches by using 24 unique search term combinations, as well as by conducting the searches from both Sydney (where the investigators are located) and Perth (through a virtual private network). Finally, given that AYAs use web-based resources and information, an important next step will be understanding what AYAs themselves think about available web-based resources in terms of appropriateness or usefulness, which was beyond the scope of the current environmental scan conducted here.

### Conclusions

AYAs diagnosed with cancer frequently turn to the internet to seek information related to their diagnosis, treatment, and psychosocial needs [24,25,76,87]. Information accessed on the internet can play a major role in AYAs’ decisions to seek care or support to address their specific needs and concerns [24,25,76,87]. Findings from this environmental scan suggest AYAs diagnosed with cancer in Australia would benefit from more tailored, evidence-based, and culturally inclusive web-based resources that are easy to locate, are provided in multiple formats (eg, text as well as audio-visual), are presented at the reading level of someone in year 8 or below and are easy to act on. While some resources describe their development as being done in consultation with survivors of AYA cancer, it is unclear to what extent a co-design approach was taken. A co-design approach would be beneficial to at least ensure the understandability, readability, actionability, and cultural inclusivity of any future resources developed.

## Appendix

Multimedia Appendix 1 Resources identified.

## Abbreviations

AYA: adolescent and young adult
NGO: nongovernmental organization
PEMAT: Patient Education Materials Assessment Tool
SHeLL: Sydney Health Literacy Laboratory

## Data Availability

The data sets generated during this study are available from the corresponding author on reasonable request.
