# Supplementary material for: Exploring Web-Based Information and Resources That Support Adolescents and Young Adults With Cancer to Resume Study and Work: Environmental Scan Study
Source: JMIR Cancer. 2024 Mar 25;10:e47944. doi: 10.2196/47944 (PMC11002739; doi:10.2196/47944)
Supplement: Multimedia Appendix 1 [file cancer_v10i1e47944_app1.docx]

**Table S1.** Resources identified through the environmental scan.

| Resource title | Publishing Source/Organisation | Country | Location in a search (page #) | Date of resources publication | Text and/or Video | Understandability, actionability and readability scores | Cultural inclusivity  (no. of criteria met/7) | Evidence-based | Co-design method used |
| --- | --- | --- | --- | --- | --- | --- | --- | --- | --- |
| *Education* | | | | | | | | | |
| Cancer in the School Community [65] | NGO: Cancer Council | Australia | Page 1 | Published: 2008  Updated: 2018 | Text | Understandability: 94%  Actionability: 80%  Readability: 10.5 | 2 | No | Unclear: describes use of interviews with stakeholders such as survivors or clinicians, but no detail on the number of interviews, or number/characteristics of stakeholders is provided. |
| University, TAFE, and Cancer Treatment [60] | NGO: Canteen | Australia | Page 1 | None provided | Text and video | *Text*: Understandability: 90%  Actionability: 100%  Readability: 11  *Video:*  Understandability: 80%  Actionability: 0% | 2 | No | No |
| High School Resources and Schedules [56] | Hospital: Seattle Children’s Hospital | USA | Page 4 | None provided | Text | Understandability: 93%  Actionability: 80%  Readability 8.5 | N/A | No | No |
| Keep Up with School [54] | Hospital: St Jude Children’s Research Hospital | USA | Page 3 | None provided | Text and video | *Text*: Understandability: 80%  Actionability: 100%  Readability: 8.5-13  *Video:*  Understandability: 60%  Actionability: 0% | N/A | No | Unclear: states “teen and young adult survivors told us what they needed” but does not describe how this consultation was conducted. |
| For Students with Chronic Health Issues [57] | NGO: Center for Web-based Education | USA | Page 5 | None provided | Text | Understandability: 77%  Actionability: 40%  Readability: 15.4 | N/A | No | No |
| School and Career [58] | NGO: Stupid Cancer | USA | Page 2 | None provided | Text | Understandability: 85%  Actionability: 60%  Readability: 13 | N/A | No | No |
| Back to Education after Cancer [70] | NGO: Teenage Cancer Trust | UK | Page 1 | None provided | Text | Understandability: 92%  Actionability: 60%  Readability: 10.6 | N/A | No | Unclear: quotes from interviews with AYAs are provided, but it’s unclear to what extent consultation with AYAs informed development of the information presented. |
| *Employment* | | | | | | | | | |
| Young People Going Back to Work with Cancer [64] | Government body: Government of South Australia | Australia | Page 1 | Published: ummari  Updated: 2022 | Text | Understandability: 83%  Actionability: 80%  Readability: 10.4 | 0 | No | No |
| Going Back to Work [61] | NGO: Canteen | Australia | Page 3 | None provided | Text and video | *Text*: Understandability: 90%  Actionability: 100%  Readability:  *Video:*  Understandability: 80%  Actionability: 0% | 2 | No | No |
| Employment and Cancer Treatment [62] | NGO: Canteen | Australia | Page 3 | None provided | Text and video | *Text*: Understandability: 90%  Actionability: 100%  Readability:  *Video:*  Understandability: 80%  Actionability: 0% | 2 | No | No |
| Cancer and Careers [53] | NGO: Cancer and Careers | USA | Page 1 | None provided | Text | Understandability: 85%  Actionability: 60%  Readability: 9-16 | N/A | No | No |
| How Your Employment May be Affected by Cancer [73] | NGO: TrekStock | UK | Page 5 | None provided | Text and video | *Text*: Understandability: 100%  Actionability: 100%  Readability: 14.7  *Video:*  Understandability: 80%  Actionability: 90% | N/A | No | No |
| Going Back to Work After Cancer Treatment [72] | NGO: Young Lives vs. Cancer | UK | Page 3 | None provided | Text | Understandability: 92%  Actionability: 80%  Readability: 9.7 | N/A | No | No |
| What should be considered when planning a return to work after cancer? [68] | NGO: Shine Cancer Support | UK | Page 4 | Published April, 2016 | Video | Understandability: 60%  Actionability: 50% | N/A | No | No |
| Returning to Work After Cancer Treatment [71] | NGO: Teenage Cancer Trust | UK | Page 2 | None provided | Text and video | *Text*: Understandability: 90%  Actionability: 100%  Readability: 9.2  *Video:*  Understandability: 80%  Actionability: 0% | N/A | No | No |
| *Education and Employment* | | | | | | | | | |
| Returning to School or Study [63] | NGO: Canteen | Australia | Page 1 | None provided | Text and video | *Text*: Understandability: 90%  Actionability: 100%  Readability: 8.8  *Video:*  Understandability: 80%  Actionability: 0% | 2 | No | No |
| Thinking ahead [67] | Hospital: Peter MacCallum Cancer Centre | Australia | Page 5 | 2017 | Text | *Text*: Understandability: 90%  Actionability: 100% | 0 |  | Unclear: describes youth participation but extent and methods unclear. |
| Returning to Work or Study [66] | NGO: Leukaemia Foundation | Australia | Page 1 | Updated June, 2020 | Text and video | *Text*: Understandability: 80%  Actionability: 100%  Readability: 8.5-13  *Video:*  Understandability: 80%  Actionability: 100% | 0 | No | No |
| School and Work During Cancer [52] | NGO: Cancer.net | USA | Page 1 | Published June, 2019 | Text and video | *Text*: Understandability: 90%  Actionability: 100%  Readability: 9-11  *Video:*  Understandability: 80%  Actionability: 0% | N/A | No | No |
| Adulting with Cancer [55] | Hospital: St Jude Children’s Research Hospital | USA | Page 2 | None provided | Text | Understandability: 80%  Actionability: 100%  Readability: 9.7-15 | N/A | No | Unclear: states “teen and young adult survivors told us what they needed” but does not describe how this consultation was conducted. |
| Work, School, and Finances [59] | NGO: Leukemia and Lymphoma Society | USA | Page 3 | None provided | Text | Understandability: 92%  Actionability: 80%  Readability: 11.8 | N/A | No | No |
| Returning to work or school after cancer treatment [74] | NGO: Leukemia and Lymphoma Society of Canada | Canada | Page 3 | None provided | Video | Understandability: 90%  Actionability: 100% | N/A | No | No |
| School and Work Counselling [75] | NGO: Pediatric Oncology Group of Ontario | Canada | Page 5 | None provided | Text | Understandability: 80%  Actionability: 80%  Readability: 8.5 | N/A | No | No |
| Study, University, and Work [69] | NGO: Blood Cancer UK | UK | Page 4, | None provided | Text and video | *Text*: Understandability: 90%  Actionability: 100%  Readability: 9-11  *Video:*  Understandability: 80%  Actionability: 0% | N/A | No | No |

^a^NGO: nongovernment organization,

^b^Because no resources directly used language that was strengths-based and respectful of Aboriginal and Torres Strait Islander peoples, we indicated this criterion was met if there was at minimum an acknowledgment on the web page recognizing these populations.
